# New York Pathological Society

**Published:** 1859-12

**Authors:** E. Lee Jones


### ART. II.—*New York Pathological Society.*

REGULAR MEETING, Oct. 12. 1859. JOHN M. C. DALTON, M. D., President, in the Chair.

(Reported for the Journal by E. LEE JONES, M. D., Secretary.)

### FOREIGN BODY IN LEFT BRONCHUS. TRACHEOTOMY.

Dr. CONANT presented a small piece of apple, which was removed from the trachea of a child, with the following history:—

On the 28th September, a little boy, six and a half years old, named Francis P. Calahan, while eating an apple, became suddenly choked, and fell to the floor. His father immediately picked him up, and ran his finger down his throat, and felt a piece of apple, which he crowded down. The child was at once relieved, and began to breathe quite freely. A few moments after, however, the breathing became difficult, when he was brought to the Demilt Dispensary, and placed in the surgical room, over which Dr. C. had charge.

The case was very carefully examined, and the œsophagus explored with a sponge probang, so as to be sure that no portion of the apple remained there. At this time the air was passing into both lungs, although the breathing had become quite difficult.

An emetic was then administered, by which considerable quantities of apple was brought up, but without any relief. Dr. C. then proposed to open the trachea. To this the father objected, saying that the child had had inflammation of the lungs and asthma, and was not breathing then with any more difficulty than he had on previous occasions.

On the Friday following, the little patient was attacked with great difficulty of breathing; and suffocation threatening, he was again brought to the Dispensary; and, on examination, it was found that the left lung had almost entirely ceased to move, no air passing into it, while the right lung was quite active, indicative, as Dr. C. supposed, that the piece of apple had lodged in the left bronchus.

The lips were blue, the countenance exceedingly anxious, the child almost unable to swallow, and, to all appearances, could live but a short time. Dr. C. then told the father that the trachea must be opened, to save the life of the child. The father again objected, but thinking that the child might soon die, the parents went and had a daguerreotype taken of the little sufferer.

Upon further consideration, the parents finally consented to have the operation performed, which was done by Dr. C., at 4 o'clock, P. M., Sept.

30th. There were present, Drs. Cummings and Dunster, and Messrs. Holden, Hagan, Walker, and Steele, medical students.

Three veins were ligatured before reaching the trachea, when all hemorrhage had ceased; the second, third, and fourth rings of the trachea were divided; the trachea forceps were introduced, and first turned upward, in order to remove anything that might be lodged above the opening. They were then turned downward to the bifurcation of the trachea, but nothing could be seized. A silver wire, bent in the middle, was then passed into the trachea, and down the right bronchus, with the hope of bringing it in contact with the border of the foreign body, and thus dislodge it, but also without success. The child was then suspended by the heels, and the chest forcibly compressed. This attempt was also in vain. A double canula was then inserted, and directions given that the child should be carefully watched.

The effect of the chloroform soon passed away, and he breathed with less difficulty than before the operation. There was no sensible change, however, until the following Sunday morning, when the attention of the parents was called to the child by what they termed a slight explosion from the instrument, when, upon examination, this small piece of apple was found lying by the side of the canula. Five hours after, the instrument was removed, and the child very soon began to breathe through the larynx. Since that time the wound has gradually closed up; and now but very little air passes out through the opening in the trachea, and the child is playing around the house with his brothers, as the parents say, suffering less than for two years, with his asthmatic breathings.

### FIBROUS TUMOR OF THE UTERUS.

Dr. FINNELL presented a specimen of *fibrous tumor of the uterus*, which had grown from different parts of the organ, so as almost completely to obliterate its cavity. The ovaries, in consequence of inflammation, had become adherent to the posterior surface of the organ.

The specimen was taken from the body of a woman who was beaten, or supposed to have been beaten, to death by her husband. She was very intemperate in her habits, and was the mother of three children. While in a state of intoxication she had a quarrel with her husband, who beat her over the head with a stick, and death followed soon after.

On making the autopsy, while engaged in taking out the uterus, the gentleman who was removing the calvarium, called his attention to what he supposed to be a fracture of the skull; but it turned out to be a fissure produced by the forcible manner in which the skull cap was removed by the chisel and hammer before the bone had been sawn through all around.

This state of things is of very frequent occurrence, and is sometimes of immense importance in a medico-legal point of view, and very often makes a strong item of evidence in a case. The fracture that is produced by violence before death, is always distinguishable from that made during an autopsy, by extravasated blood around and through the fissure; while, on the other hand, the break is clean.

### TUBERCULOUS LARYNX.

Dr. Finnell presented a second specimen of a *tuberculous larynx*, for a candidate.

### INJURY OF RIBS—NECROSIS—REMOVAL OF BONE.

Dr. Finnell lastly related the case of a boy, nine years old, who sustained an injury of the right side of the chest, from being run over by a wagon. The gentleman who was called to see the case, pronounced the injury to be a fracture of the fifth and sixth ribs, applied a firm body-bandage, and directed the patient to remain quiet in bed. In a few days the doctor called again, but finding the boy very comfortable, left him for a week, at the end of which time he found him well enough to be about. The bandage was still firmly applied to the chest; everything seemed to be going on so finely that he did not think it worth while to see the child again, and simply left orders with the mother that the bandage should not be removed until the end of four weeks. In about four weeks he called again to present his bill, and finding the bandage still applied, left directions to have it removed within three or four days.

When the mother came to remove the bandage and look at the parts, she thought it was not yet healed over, and sent for the doctor again. When the doctor arrived, he found, to his surprise, *the black and necrosed ends of the ribs protruding through the flesh.* Never having heard or seen anything of the kind, he requested me to visit the case with him, and he (Dr. F.) removed the dead portions with the bone-nippers. The portions cut off were very smooth, showing that the case was not one of fracture, but separation—disarticulation of the fifth and sixth ribs from the costal cartilages.

The cause of this trouble was very evident; the bandage was applied so snugly that the separated ends were forced through the integuments, thus becoming necrosed. Had no bandage been applied, the case would have progressed far more satisfactorily. The point of surprise in the case was, that with such a state of things going on, the little patient should experience no pain, and was able to bear the bandage applied such a length of time without a murmur, notwithstanding there was more than half an inch of rib protruding.

### IMAGINED INTESTINAL PARASITE.

Dr. Jno. C. Dalton related the history of a specimen that was brought to him that morning and, he regretted to say, taken away again.

A gentleman brought me, this morning, in a bottle of alcohol, a mass that had the appearance of a very thick, fat leech, about two inches in length, evidently some organized structure, evidently also an animal, which the gentleman said had been discharged from the intestinal canal of a patient some two or three months previous, at which time it was alive; since then it had been preserved in alcohol.

It was nearly cylindrical in shape, one extremity tapering almost to a point, and the under surface was mottled with grayish slate-colored spots or blotches.

The account which he gave was, that the patient who passed this worm had been suffering from some affection of the bowels; constipation he believed; had taken an injection, and immediately afterwards something attracted his attention by moving about under his clothes, and on making a search, found the animal in question.

It was indeed to him a very unusual object for an intestinal parasite. He was certain he never saw anything of the kind before; but on examining it a little more closely, it turned out to be a slug, one of the snails without shells. Its specific characters, as nearly as they could be made out, showed it to belong to a kind that goes under the name of *limax flavus*, which is the only one he knows of in that region of country. The gentleman who brought the specimen was very unwilling to believe that it had not been passed from the intestinal canal, as the patient had supposed; but taking everything into consideration, he (Dr. D.) could not help thinking that it belonged to a class of which a great many similar cases were recorded, of animals being discharged apparently from the intestinal canal and stomach, which never had gained an entrance into the body. And if this yellow slug was really thus discharged, it would be an unique case. The man who supplied this specimen was entirely positive in his belief that the animal had been discharged from his intestines, had attributed its presence to the fact that previously he had been in the habit of drinking of lake water, and supposed that either the animal itself, or the egg from which it had been formed, was thus introduced.

On examining it, it is found to belong to a perfectly well defined species, and more than that, it does not live in the water. It feeds upon vegetables, is usually found in gardens, and is tolerably common in the gardens about New York. His (Dr. D.'s) explanation of the matter is, that the person—a porter in a hotel—was in the habit of going to market, and had been engaged in getting vegetables, cabbages, perhaps parsley or the like, and this slug was brought to the house upon some of these, and had been accidently introduced into his clothes. He supposes, exactly parallel to this are the stories of those persons who are said to vomit lizards and strange animals. He finds, if the case is properly investigated, they can never state positively that such has been the case. It resolves itself into this: a person vomits, and immediately after sees a lizard upon the grass, and concludes, in as much as it lies in the vomit, not having seen it before, that it came from the stomach. Here the animal was found under the clothes, and the conclusion was that it came from the rectum.

### EPILEPSY—TREPHINING.

Dr. Sayre presented a *button* of bone, removed from the vertex of the skull of a man, by the trephine, for the purpose of curing epilepsy, complicated with mania. The history of the case is as follows:

A man, some eight months since, received a blow on the vertex of his skull by the fall of a brickbat. The injury was accompanied by the flow of a considerable amount of blood. He found him a few days ago in the cells of the hospital, confined for mania. For the last two months, together with repeated attacks of convulsions, he also suffered from delirium.

He had been subjected to all kinds of medical treatment, but it availed nothing. Having learned from his wife that he had received a blow upon the head sometime previous, a careful examination was made; and a concavity on the vertex, about half an inch in length, was detected, in which the fore-finger readily dropped. From this fact, it was thought possible that a portion of the internal table might be driven down upon the brain. He also traced the origin of the pain to that portion of the head. A consultation being held, it was determined best to trephine. He performed the operation to-day. Notwithstanding the trephine was applied directly over the longitudinal sinus, there was not the slightest amount of hemorrhage; the dura mater was left perfect. The button of bone being removed, there was discovered a slight depression on external surface, and a fissure passing through to under surface; but there was no corresponding depression. The dura mater at that point was covered with small spiculæ of bone; one portion of the button of bone removed was eburnaceous, the diploe being entirely absent.

In conclusion he remarked, that the history of those cases of epilepsy for which trephining had been resorted to, was attended with better results than any other treatment for that affection.

Dr. Bibbin asked whether, in these cases of epilepsy resulting from a fall, if a person necessarily receives the shock upon the head, or whether, if he strikes his feet foremost, epilepsy follows?

Dr. Sayre supposed that concussion of the brain was probable from a fall upon the feet.

Dr. Bibbin stated that he was sent for to see a woman who, in attempting to escape from her husband, leaped from a fourth-story window, and alighted upon her feet. Epilepsy followed the accident, which was evidently the cause, as she had not suffered from anything of the kind before. It was the recollection of this case that prompted him to ask the question. In relation to the facts, he stated that he only depended upon the testimony of those who resided in the house.

---

Regular Meeting, October 26, 1859. A. C. Post, M. D. President *pro tem.*

DISEASED KNEE JOINTS.

Dr. Bauer presented two specimens of disease of knee joint. The history of the first was as follows:

A healthy boy, about 14 years of age, was struck with a stone against the shin, immediately below the knee joint; intense periostitis ensued, which was neglected; the result of all which, was the specimen presented. Around the seat of injury the periosteum was entirely destroyed, and peeled off from the bone. There were two perforations of the joint; one was exceedingly interesting, viz. through the cartilage of the head of the tibia; the other being behind the right condyle of the femur. There were also manifest, frightful changes, appertaining to the presence of matter in the joint; the cavity being filled, and the surrounding tissues infiltrated, with a gelatinous mass. The disease was irreparable, and amputation was found necessary, the child's general health becoming very much enfeebled. In consequence of the extensive injury to the soft parts, it was necessary to amputate high up.

In relation to the second case, Dr. B. remarked that it was one of great pathological interest. The history of the case, said he, is of a somewhat lengthy nature; I will, however, give it in outline.

A somewhat enfeebled tailor was admitted a patient in the Long Island College Hospital. All that he presented pathologically, was the remains of erysipelas of the face; he had no fever; in fact he seemed to be free from any constitutional disturbance, yet he would become delirious, at times violently so, besides having a considerable tremor of the muscles. In a word, all the peculiarities of delirium tremens presented themselves; but the person being one of known temperate habits, entirely excluded that diagnosis. The next most likely affection it was thought to be, was uræmic poison: the urine was accordingly examined. There was found a moderate quantity of albumen, besides some casts which clearly showed a diphtheritic degeneration of the medullary substance of the organ. He was treated accordingly, and the delirium left him in 36 hours. His recovery proceeded to a certain extent, when suddenly his knee joint was filled with matter. Up to that time, the man was getting along well enough, and it was exceedingly difficult to find out the cause of the trouble.

He suffered from no pulmonary pain or uneasiness, and, even when the joint became filled with matter, he did not complain. This collection of matter must have formed very rapidly, as I saw him within ten or twelve hours of its occurrence, and he did not give the slightest indication of trouble in that locality. The matter was twice removed by the trocar, without any difficulty occurring, or inflammation supervening.

Soon after, he became very decidedly enfeebled; his skin became very pallid; in fact, presented all the characteristic appearances of a person with a superabundance of white corpuscles. His blood was examined, and more than fifty per cent. of white corpuscles existed. This remained throughout the rest of the disease (he died of course).

On post-mortem examination, we found, besides degeneration of the kidney, and some disease of the lungs, this condition of the knee joint. I need not state that the disease of the knee joint went on. I have never heard of such a case, where the white corpuscles existed in such abundance. The blood was first taken from the affected leg, the seat of disease, and where there appeared such an abundance of white corpuscles; it was thought to be due to the presence of pus, when it was taken from a different part of the body. The destruction of the joint will be seen to be very considerable and extensive; the cartilage is all gone, and the synovial membrane, for the most part, also.

### BLOOD-CLOT IN FOURTH VENTRICLE.

Dr. B., next presented a specimen of blood-clot, found in the fourth ventricle. It had brought on sudden dea h; and, as the woman had been beaten by her husband, it was supposed that the death was the result of this violence, but this was not confirmed.

### RETROVERSIO UTERI CAUSED BY AN ABSCESS—PERFORATION OF THE RECTUM —PERITONITIS—DEATH.

He next presented specimens consisting of the rectum, womb, bladder, and a portion of the small intestine, which were removed from a woman who had been in a delicate state of health for a long time; still she was able to get about and do her house-work. She suffered from trouble of the womb, to remedy which she applied to a physician; who made a correct diagnosis, pronouncing it to be retroversion.

An attempt was made by the physician to put the organ in place; and, if I understood her rightly, the finger was introduced into the anus, another into the vagina, and the basis of the organ was tried to be raised. The operation was exceedingly painful to her; so much so, that she desired to be left alone unrelieved. She went home, and was attacked by a very violent peritonitis; the treatment which was instituted proved of no avail, and she died six weeks afterwards.

We found, on post-mortem examination, an abscess immediately in front of the basis of the womb, which seems to have pressed the organ constantly down. How long this collection of matter existed, we are of course unable to say; but from the great changes that have taken place in the surrounding parts, we infer that it must have been a good while, as is usual with these abscesses. The rectum was perforated, and pus was discharged in considerable quantity from it. This state of things showed itself during my treatment of the case; and the peculiar character of the stools led to a microscopical examination of the matter; when, by the presence of such an amount of pus globules, the existence of an abscess was clearly made out, and the tumor which was detected immediately behind the bladder and to the right side, was supposed to be its seat. The lower wall of this abscess is the posterior wall of the bladder, and the anterior wall of the uterus, the basis of the organ, and the anterior wall of the rectum. Above, the abscess was closed by the small intestines, which were closely and firmly agglutinated by plastic material—so firmly, indeed, that the patient was able to pass nothing but matter from the rectum, for the last four weeks of her life. All efforts to relieve her, in this particular, were fruitless.

He exhibited portions of intestines, in order to demonstrate how extensive and serious was the damage done by the exudation; at one point the opposite sides were adherent to each other.

He was not certain of the cause of the trouble; he thought it possible, however, that it might be due to the presence of tubercles, as a mass of that material was found attached to a portion of intestine, and also a few tubercles were found scattered in other places over the peritoneum.

### LIVER, INTESTINES, AND OMENTUM AFTER PERITONITIS.

He next presented the liver, a portion of intestine, and omentum, which were taken from the body of a man who had been beaten and kicked by a woman. The examination was made at the request of the coroner.

The deceased was in good health for a period of ten days after the occurrence, when he was somewhat suddenly taken down, and died in two days.

On making the post-mortem examination, a most intense peritonitis was found to exist throughout the whole abdominal cavity, involving nearly the entire peritoneal surface of the liver, and covering it with false membrane. The parenchyma of the organ was also involved in the inflammation.

The cause of all this trouble was, two perforations of the intestines, one of which was situated near the margin. A portion of the omentum was also exhibited, to give an idea of the intensity of the inflammation, and the quantity of plastic material effused.

### REPRESENTATIONS OF CANCER IN WAX.

Dr. B. next presented four specimens of wax preparations, consisting of a cancerous liver, cancer of the lip, cancer of small intestine and mesenteric glands (the intestine laid open), and melanotic cancer of the cephalic vein of the left arm, which he prepared himself by casts from the morbid specimens, first making one of plaster of Paris, and afterwards of wax, and giving them all the color required. By these means they were rendered true to the original, and formed an excellent substitute for the specimen in its fresh state.

### PERIGOFF'S AND SYME'S STUMPS.

In this connection he exhibited three casts of stumps, one of which was the result of Syme's operation, and the other two of Perigoff's. The latter he was indebted to the kindness of Prof. Pancoast, of Philadelphia, for the opportunity of exhibiting. He only wished to compare the results of the two operations. In regard to the usefulness of the stump, he thought that one answered as good a purpose as the other, with the exception that Perigoff's operation leaves the limb longer; and in consequence of the increased bulk at the extremity, mechanical appliances could not be as well made as in Syme's stump. This, he said, was the opinion of those who were in the habit of making such apparatus.

Last in order, the Dr. presented a foot of a very peculiar appearance, and gave the following history :—

The patient presented himself about ten days before at my office; and I candidly confess I did not know what to make out of the foot. It was easy to see from the appearance presented, that there was dislocation of the first phalanx of the great toe at the metatarsal articulation; but the peculiar appearance and the position which the ankle assumed (the heel being projected three quarters of an inch more backward than its fellow), puzzled me not a little.

On questioning the mother, I ascertained that ten years ago, the child, being two years old at the time, fell down stairs, and remained for a considerable time with the heel of the right foot caught in the balustrades in such a way that the foot turned almost upwards, and, according to her statement, a subluxation took place. Inflammation ensued; this produced reflex action upon the flexor muscles of the foot, the tibialis anticus and peroneus muscles being very strongly flexed. The arch of the foot is entirely lost, and the *os naviculare* is sunk down as in the ordinary flat-foot. I have divided the flexor muscles of the foot and large toe, and I am happy to say, I have replaced the form of the foot entirely; which success I owe in a great measure to the kind co-operation of my friend Dr. Sayre.

Dr. Sayre, in relation to the second specimen presented by Dr. Bauer, said, that it was peculiar in respect to the structure of the femur, which was markedly of an eburnaceous character: so dense and so compact was its structure, that there were scarcely any cells present in the bone; and yet a little beyond you find the joint involved in caries.

Dr. Post referred to a somewhat similar case, which he had occasion, several years ago, to amputate for caries of the knee-joint, and the bone above, for several inches, was so hard that it could hardly be sawn through.

Dr. Sayre asked if the smallness of the medullary canal threw any light on the nature of the disease, whether or not there would be a tendency to cut off the supply of blood to the part, and so give rise to the trouble?

Dr. Post said, that he would rather regard this eburnaceous condition of the bone as the consequence, than the cause, of the caries.

### URIC-ACID CALCULI IN AN INFANT.

Dr. Bibbins presented two small uric-acid calculi, which came from a child, with the following history:—

"I first saw the child," said he, "on the 12th of August last, and it was then not quite three months old. The nurse, under whose charge it was, had obtained it from the mother, who was confined in the Lying-in-Asylum. It was fed by artificial means. The nurse stated that the child had passed, in all, seven calculi, four of which she lost. These were discharged in one day, and the little one seemed to suffer no appreciable pain."

The doctor prescribed fomentations, warm baths, and internally a mixture of syr. ipecac. and spirits mindereri. The nurse came to him again on the 19th of the month (Oct.)—the child being more than five months old—to report the little one entirely recovered.

### LUMBAR ABSCESS.

Dr. Sayre next presented a specimen of so-called lumbar abscess; but in that particular instance, he thought it more fitting to denominate it a periostitis of the lumbar vertebræ.

The patient was a man, 32 years of age, who, up to a year before, enjoyed strong, vigorous health, with no symptoms of any strumous disease in himself or family.

The first indication of trouble which he experienced was a sense of lameness and weakness in the lumbar region, accompanied with more or less general debility, which continued until four months ago.

When he entered the hospital, there was then found to exist a small abscess on the left side of the lumbar vertebræ, near the situation of the sacro-iliac synchondrosis, and also a prominent tumor on the right side, nearly in the same situation.

Within two or three weeks after his admission, the doctor said it grew quite rapidly, and it was explored and opened, but only discharged a quantity of fœtid gas. Two or three days after this, pus was discharged quite freely. I saw the patient for the first time only two or three days since, and on introducing the probe, found that it passed in, several inches, in various directions. The posterior surface of the ileum was denuded of its periosteum. The patient's general condition was too much exhausted, and it was determined best to let him alone; and he gradually sank, and died soon after of diarrhœa.

On making the post-mortem examination, we ascertained that the periosteum was covered with osteophytes; on almost all the vertebral bones in the vicinity which were not entirely denuded; and, in close connection with these growths, necrosis was observed. There was quite a projecting point, half an inch in length, at right angles with the sacrum, which penetrated the rectum. At the junction of the ileum and sacrum, on the right side we found the greatest amount of disease; and at that point the abscess had perforated the fascia of the psoas and iliacus muscles, but the matter had not burrowed far enough forward to show itself on the thigh before death, only arriving as far as half an inch below Poupart's ligament.

There was no evidence of tubercles in any part of the system; his intestines, liver, lungs, in fact, every organ of body was perfectly well in that respect. This specimen goes very strongly to disprove the views entertained by Dr. Gross in relation to hip-joint disease and kindred affections.

### CAPUT FEMORIS REMOVED FOR MORBUS COXARIUS.

Dr. S., next presented a specimen of the head of a femur, which he removed from a little child about 11 years old. The operation was performed on last Thursday. The child had been suffering from the disease for four years; and, not being in the hands of a regular practitioner, nothing was attempted for his relief. I saw him for the first time on last Saturday a week, and found him extremely enfeebled, and attenuated to the very last degree, pulse 165, lying on his back, with legs drawn up very much, one at an angle of 60 degrees. A portion of the trochanter major was denuded. In addition to all this, there existed four or five abscesses in the vicinity, which freely communicated with the hip-joint.

After a consultation, it was resolved to attempt the removal of the head of the bone. The patient was put under the influence of chloroform, and a careful examination made. The head of the bone was found to be denuded, and gave the sensation of grating against bare bone; but from the very peculiar position assumed by the limb, it was doubtful to his mind whether the acetabulum might not be perforated; but, not being able to examine it to his satisfaction, he was unable to make up his mind definitely in relation to the point. After the delay of a few days, in order to try if possible to get up the strength of the patient, the operation was performed, as the only chance left for saving his life.

On removing the head of the bone, he found it fastened through an opening into the acetabulum; and, on withdrawing it, a large quantity of green-colored, fœtid pus escaped. The opening into the acetabulum corresponded in size and shape exactly with the portion of bone that occupied it, so that it formed a complete plug to prevent the escape of the confined matter; as far as the finger, passed through this opening, could reach, it was found that the internal surface of the ileum, ischium, and pubis was denuded of periosteum, and evidently in a state of caries.

The periosteum, being peeled off from the surface of these bones, formed a cavity or bag for containing the pus. All the carious portions were removed by the bone-forceps, leaving almost nothing behind but the wing of the ileum. During all this operation, the periosteum and peritoneum were not interfered with, and formed the only partition between the pelvic cavity and the external world. This was done on Thursday, and the pulse, previously to the operation, was 160 when the child was asleep. The chloroform was administered while he was in that state, so that the child did not wake up till after the operation. Dr. Jones superintended the administration of the anæsthetic, and too much credit could not be given for the manner in which it was done.

The evening following the operation, the pulse fell to 120, and remained so till the Sunday following, when it had decreased to 112. The patient was dressed for the first time that day, and presented a very favorable appearance. The wound was nicely washed and injected, and the doctor began to entertain strong hopes of recovery. Since that time the patient has been going on tolerably well, and notwithstanding everything was done for the little fellow, in the way of nourishment, he feared that he had not vitality left, to repair the damages.

The doctor explained the perforation of the acetabulum, by referring to the fact that, eight months before the operation, the patient had fallen from the bed, and struck upon the trochanter of that side. The head of the bone, being thus driven through, peeled off the periosteum, and formed the pocket for the pus.

Dr. BAUER remarked that the case was not a fair one for operation, that its performance was with the view only of allowing a free exit to matter, and rendering the last hours of existence more tolerable. He had watched the case carefully, and was satisfied that the patient was far more comfortable after the operation, than he had been during the eight months before. He maintained, that the operation, in the case referred to, demonstrated the fact that it could be performed with benefit to the patient, not only when the head of the femur was involved, but also the acetabulum. He stated, that the number of operations for hip-disease has reached the handsome number of ninety-two, and almost *fifty per cent.* recovered. Very little blood was lost, hardly more than half an ounce at any time. In eight cases, he had no occasion whatever to ligate an artery. In Dr. Sayre's case, one small vessel was tied, and this would not even have been required, were it not for the extreme feebleness of the child.

The question presented itself to his mind, in that connection, how it came, that abscesses and large deposits of purulent matter could remain and be manufactured in the body for months, and not produce that disease denominated pyæmia. He thought it was time to take up the matter and study it more closely, especially as the latest work on Surgery had revived all the old doctrines in relation to that form of disease, notwithstanding many of the theories had been exploded twenty-five years ago.

